# Complex Aerosol Characterization by Scanning Electron Microscopy Coupled with Energy Dispersive X-ray Spectroscopy

**DOI:** 10.1038/s41598-020-65383-5

**Published:** 2020-06-04

**Authors:** Anders Brostrøm, Kirsten I. Kling, Karin S. Hougaard, Kristian Mølhave

**Affiliations:** 1 Technical University of Denmark, DTU Nanolab – National Centre for Nano Fabrication and Characterization, Fysikvej, Building 307, 2800 Kgs Lyngby, Denmark; 2 National Research Centre for the Working Environment, Lersø Parkallé 105, 2100 Copenhagen, Denmark; 3 SAXOCON A/S, Bredevej 2D, 2830 Virum, Denmark

**Keywords:** Occupational health, Sustainability, Environmental sciences, Nanoscience and technology, Techniques and instrumentation

## Abstract

Particulate matter (PM) air pollution is a central concern for public health. Current legislation relies on a mass concentration basis, despite broad acceptance that mass alone is insufficient to capture the complexity and toxicity of airborne PM, calling for additional and more comprehensive measurement techniques. We study to what extent scanning electron microscopy coupled with energy dispersive X-ray spectroscopy (SEM/EDS) can be applied for physicochemical characterization of complex aerosols, and investigate its potential for separating particle properties on a single particle basis, even for nanosized particles. SEM/EDS analysis is performed on impactor samples of laboratory generated aerosols, consisting of either NaCl, Halloysite fibers, soot-like Printex90 agglomerates, or their combination. The analysis is automated and performed as EDS maps, covering a statistically relevant number of particles, with analysis times of approximately one hour/sample. Derived size distributions are compared to scanning mobility particle sizer (SMPS) and electric low-pressure impactor (ELPI) results. A method is presented to estimate airborne number concentrations and size distributions directly from SEM results, within a factor 10 of SMPS and ELPI outcomes. A classification scheme is developed based on elemental composition, providing class-specific information with individual particle statistics on shape, size, and mixing state. This can identify primary particles for source apportionment and enables easy distinction between fibrous and dense particle classes, e.g. for targeted risk assessments. Overall, the SEM/EDS analysis provides a more detailed physicochemical characterization of PM than online measurements, e.g. SMPS and ELPI. The method has the potential to improve assessments of PM exposure and risk, and facilitates source identification, even without prior knowledge at sampling.

## Introduction

Particulate matter (PM) is present in both ambient and occupational settings and constitutes a major public health concern. Epidemiological and toxicological studies have linked inhalation of PM to a broad spectrum of acute and chronic health effects, among others in the cardiovascular, respiratory and immune systems as well as to cancer and reproductive effects^1–4^. Not surprisingly, PM exposure is recognized as a major contributor to the global burden of disease^5,6^. Globally, exposure to outdoor PM_2.5_ was estimated to account for 4.2 million deaths and 103.1 million disability-adjusted life-years in 2015^7^.

Despite the well-known problem of PM pollution, the related exposure and risk assessments are exceptionally challenging, due to the complex nature of air pollution particles. The overall number concentration can vary from thousands to millions per cubic centimeter, while each individual particle can vary in shape, composition, mixing state, and orders of magnitude in size. Additionally, particles are in constant equilibrium with their surroundings. Therefore the physicochemical properties of individual particles will change throughout their airborne lifetime.

Current exposure limits and guidelines are mass based. In ambient environments PM is regulated by mass concentrations of particles with aerodynamic diameters below 2.5 and 10 µm (PM_2,5_ and PM_10_, respectively). In the occupational setting, exposure limits for mass concentrations are set for inhalable and respirable particle fractions, with lower exposure limits for specific particles with known adverse health effects. In the past, mass has proven a useful and simple dose metric in the association of PM exposure relative to adverse health effects. However, mass fails as dose metric for aerosols dominated by small particles, since these do not contribute significantly to the overall mass^1,8^, though they can still contribute to toxicity due to their high surface area to volume ratio^9^. The mass metric paradigm also comes up short for insoluble high aspect ratio fibers and highly reactive particles^9–14^, as such particle characteristics may be associated with substantial risks even at low PM mass exposure. Oppositely, risk may be overestimated for exposures dominated by harmless particles e.g. soluble salts^15^. It is therefore broadly accepted that mass is not the ideal metric for risk assessments^16^. This brings a need for additional descriptive measures. Particle surface area and reactivity, shape, size, number, composition, and mixing state are among the most important properties^12,15,17,18^, but none of these particle properties are adequate descriptors on their own and probably a combination of metrics are needed^17,18^. This highlights the importance of detailed physicochemical characterization of particle populations to supply the most relevant dose metrics for risk assessments and to help identify aerosol sources for preventive measures.

Particle size distributions (PSD) can be measured with high time resolution by a wide range of commercially available instruments, including the Electric Low Pressure Impactors (ELPI), Diffusion Chargers (DC), Scanning Mobility Particle Sizers (SMPS), Condensation Particle Counters (CPC), and Optical Particles Sizers (OPS). These instruments are crucial for visualizing the evolution of particle populations during work processes or during ambient conditions. However, none of these give information on particle surface area, reactivity, shape, composition, or mixing state. There are instruments available that can measure chemical composition at a high time resolution; for example the Aerosol Mass Spectrometer (AMS), though it is primarily used in atmospheric studies, since it is difficult to transport and needs careful calibration^19–21^. Furthermore, the AMS cannot give information on particle morphology or mixing state, as it measures size by time of flight (TOF), and chemical composition in the form of particle ensemble integrated mass to charge ratio spectra (m/z)^19^. A combination of several measurement techniques are therefore needed when characterizing complex aerosols, in order to overcome their individual shortcomings.

SEM/EDS analysis of impactor-collected samples has the potential to bring many of the relevant parameters on a single particle level, including size, number, shape, elemental composition, and mixing state as well as estimates on surface area^22–26^. Furthermore, aerosol particles can be sampled directly onto SEM appropriate surfaces with small portable impactors. Such samples can then be stored and transported to the microscope for analysis, making it suitable for workplace measurements. The method has been demonstrated and applied in atmospheric research to study the influence of particles on climate and weather^27–33^. Here it was also proven a useful tool for source identification, as it was capable of distinguishing soot from mineral dust, sea spray salts, and fly ash in atmospheric settings^34,35^. However, the method is to date not well established in indoor environments, workplace settings, or for particle sizes approaching the nano range, where it is primarily used qualitatively.

In this paper we explore the potential to evolve and improve the quality of SEM/EDS measurements for reliable quantification of aerosols. We evaluate how SEM/EDS can be applied in detailed physicochemical characterization to give particle properties of relevance for toxicity and source identification. We furthermore study to what extent SEM/EDS allows for automatic identification and separation of these properties on a single particle basis when analyzing complex aerosols, enabling a much more detailed characterization compared to other established aerosol instruments.

To do so, we demonstrate the characterization of aerosols consisting of several particle types, using SMPS, ELPI, and SEM/EDS analysis of samples collected by impaction, building on our previous work^36^. Four closed chamber experiments were conducted, where four different aerosols consisting of NaCl, carbon black (Printex90), Halloysite fibers, and a mixture of the three were dispersed, sampled, and analyzed to create aerosol samples that would model a very complex workplace exposure scenario. The PSD measured by SMPS, ELPI, and SEM/EDS analysis were compared, demonstrating that SEM/EDS analysis can indeed provide important and detailed information on e.g. shape characteristics, composition, and mixing state on single particle basis. Finally the possibilities and limitations in the use of impaction based SEM/EDS are discussed relative to the provided information.

## Methods

### Aerosols and choice of PM

The three types of particles were chosen as they possess significantly different physicochemical properties, each representing a unique class of real-life particles. The variation in properties also challenge the capabilities of the SMPS, ELPI, and SEM/EDS analysis.

The NaCl aerosol was generated by atomization (and subsequent drying) of a solution containing 1.0 g NaCl (purity ≥ 99.0%, Sigma-Aldrich, USA) in 500 ml nanopure water, to give particles in the size range 40–150 nm. NaCl particles were chosen as they are abundantly present in ambient air samples. Furthermore, all alkali halides are known electron beam sensitive compounds^37–39^. This poses a challenge when performing SEM/EDS analysis, where the elemental composition, shape, and size of individual particles may change during measurements. These effects can be minimized by using relatively short EDS map dwelltimes, as shown in previous work^40^.

The Halloysite aerosol was generated with a brush generator, aerosolizing a Halloysite powder (Dragonite HP™, Applied Minerals Inc., New York, US; CAS: 1332-58-7). Halloysites were chosen due to their fibrous shape, which can cause adverse health effects in humans, making a reliable quantification of high importance^10,41–43^. Halloysites are naturally-occurring hollow aluminosilicate clay mineral fibers (Al_2_Si_2_O_5_(OH)_4_. nH_2_O) with a density of 2.54 g/cm^3^, widths of 20–150 nm, and lengths ranging from 0.05 to 1.5 µm, resulting in aspect ratios (AR) of 5–10^44,45^. The fibrous shape can be an issue for the SMPS and ELPI instruments, since the charge distribution, and aerodynamic behavior of fibers can be very different from that of spheres^46–48^. The aerodynamic diameter of fibers is typically governed by their width, while their electric mobility is also influenced by their length^46^. Therefore a significant difference was expected between PSD of electric mobility diameter (SMPS) compared to aerodynamic diameter (ELPI)^49^.

The Printex90 aerosol was generated by aerosolizing a carbon black Printex90 powder (Orion Engineered Carbons, Frankfurt, Germany, CAS# 1333-86-4), also using the brush generator. Printex90 was included as a model for diesel exhaust particles and soot, making it a highly relevant compound to study. The Printex90 used in this study consists of carbon based spherical particles with a reported density of 2.1 g/cm^3^ and diameters of approximately 14 nm, which has been shown to form agglomerates ranging from <100 nm to micrometer sizes^50^. The Printex90 particles will challenge the SEM/EDS analysis because of their small size and the limited contrast of carbon in SEM images but also due to the pure carbon composition, which is similar to the carbon based Formvar substrate of the TEM grids.

### Experimental setup

**Figure 1:**
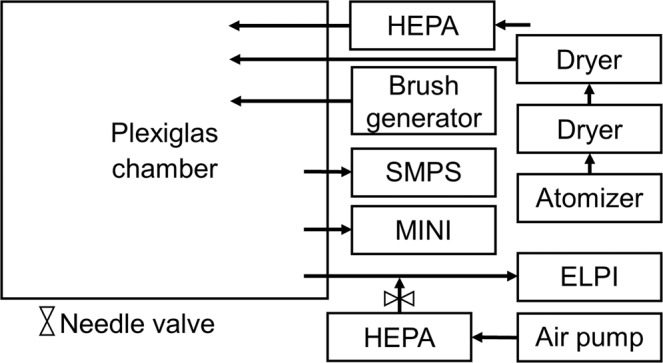
Schematic overview of the experimental setup. The HEPA filter inlet was left open to the room, in order to equilibrate the pressure as instruments pulled air from the chamber.

A set of batch experiments were conducted where each of the four aerosols were generated in a closed 0.5 m^3^ Plexiglas chamber. The Plexiglas chamber was installed with 3 inlets and 3 outlets. Polyvinyl chloride (Tygon TM) tubing (ID = 4.8 mm) was used for all connections, and was kept as short as possible, 30–50 cm, to minimize electrostatic losses^51^. One outlet was connected to a SMPS, consisting of a Classifier 3082, Neutralizer 3088, DMA 3081, and a CPC 3776 (TSI Inc., USA). The SMPS was operated in low flow mode (0.3 l/min) with an aerosol to sheath flow ratio of 1:10, detecting particles in the size range 17.5–532.8 nm. Another outlet was connected to an ELPI (Dekati Ltd, Finland). The ELPI requires a flowrate of 10 l/min, so air sampled from the chamber was diluted with HEPA filtered room air from an air pump, using a dilution ratio of 1:10. The final outlet was used to collect aerosol particles for SEM/EDS analysis, using a three stage cascade impactor (MINI). The three stages have cut-off diameters (D_50_) of 1.36, 0.59, and 0.073 µm, respectively^36^. The first impactor stage was smeared with impactor grease (Dekati Ltd, Finland) to remove large particles and ensure minimal bounce to lower stages. The second stage was equipped with a Nickel disc, as the substrate of the TEM grids were found to break upon impaction of the larger particles^36^. The final MINI stage was installed with commercially available 400 mesh nickel TEM grids coated with a 25–50/1 nm Formvar/Carbon film (Electron Microscopy Sciences (EMS), USA). All impactor samples were collected with a sampling time of 5 seconds. A schematic overview of the experimental setup is shown in Fig. 1.

Constant atmospheric pressure in the chamber was ensured by connecting a HEPA filter at one of the inlets. Another inlet was connected to a constant output atomizer model 3076 (TSI, USA), which was operated at a back pressure of 2 bar. The atomizer produced the NaCl aerosol, which passed through two diffusion dryers model 3062-NC (TSI, USA) installed with freshly dried silica gel before reaching the Plexiglas chamber. The final inlet was connected to a PALAS Brush Generator (RBG 1000, Karlsruhe, Germany), which was operated at 1200 rpm with a feedrate of 110 mm/h and a backpressure of 1 bar, resulting in a flowrate of 2.8 m^3^/h. The brush generator was loaded with either Halloysite powder, Printex90 powder, or with a mixture of the two. The brush generator consists of a piston that can be raised, feeding the loaded powder into a rotating steel brush, which carries small amounts of the powder into a flow of pressurized air, hence aerosolizing the powder.

The experiments were performed as batch experiments, where aerosol production with the atomizer, brush generator, or both was limited to short bursts. This generated high particle number concentrations, which slowly decreased over time. At several different concentrations for each aerosol, impactor samples were collected for analysis by SEM/EDS. The chamber was vented between experiments for approximately an hour until particle number concentrations dropped to levels near the initial background concentration, to ensure minimal cross contamination between experiments.

### Electron microscopy

The sampled TEM grids and nickel plates were analyzed in high vacuum mode with an Everhart-Thornley Secondary Electron (SE) detector in a Nova NanoSEM 600 (Thermo Fisher Scientific (former FEI), The Netherlands). All samples were analyzed at 10 keV, using an aperture size of 50 µm and a spot number of 3.5 with a 0.16 nA probe current. An XFlash FlatQuad (Bruker Nano, Germany) EDS detector was used to measure the elemental composition of particles by mapping the entire imaged area as detailed in our previous work^40^. Maps from the second stage of the impactor were acquired with a pixel dwelltime of 256 µs with acquisition times of approximately 4 minutes, while maps from the third stage were acquired with 128 µs dwelltime resulting in roughly 8 minutes/map, due to a larger image size. The EDS analysis was only performed on the complex impactor samples, while SE imaging was used for analysis of all samples containing a single primary particle type. To give a representative sample description, all impactor samples were analyzed by acquiring a series of images or maps in a straight line going through the center of impaction, according to the method described by Brostrøm *et al*.^36^. Previously the imaging routine was only verified for the 3^rd^ impactor stage, but as we observed similar deposition patterns on the 2^nd^ stage in this work, we chose to apply the method here as well. A fresh TEM grid and Ni disc were also investigated to ensure no particles were recognized on clean samples. Images of the clean samples are presented in Fig. 1 in the Supplementary Material. Here it was found that particles with an equivalent circular diameter below 400 nm could not be distinguished on the rough surface of the Ni disc, while the TEM grids allowed detection of particles down to ca. 20 nm.

The ESPRIT 2 software (Bruker Nano, Germany) was used for analysis of all map data, while a custom python 3.6 code with the openCV package^52^, was used to analyze SE images (available on request). All maps and images were segmented with a manually set global threshold, to distinguish particles from the substrate. Particles touching the edge of the frame were excluded from the analysis. The area (A), perimeter (P), length (L), and width (W) were determined for each individual particle. The length and width were determined by fitting the smallest freely rotating box around each particle, with the longest dimension taken as the length, and the shortest taken as the width. From these measures the equivalent circular diameter (D_eq_ = 2√(A/π)), aspect ratio (AR = L/W), and circularity (Ci = 4πA/P^2^) were calculated. In the remainder of this work, all SEM particle sizes are reported as D_eq_. For EDS analysis, the spectra from each pixel within the contour of a single recognized particle were summed and analyzed as one spectrum. The bremsstrahlung X-ray contribution was accounted for using the SEM fitting option in ESPRIT with relevant fitting areas identified automatically. The Cliff-Lorimer quantification model was used for quantification, as it ignores most matrix interactions and is therefore suited for thin electron transparent samples^34,40,53,54^. Maps acquired on the Ni disc samples were quantified with the standardless P/B-ZAF method, as matrix interactions could no longer be ignored.

## Results and Discussion

**Figure 2:**
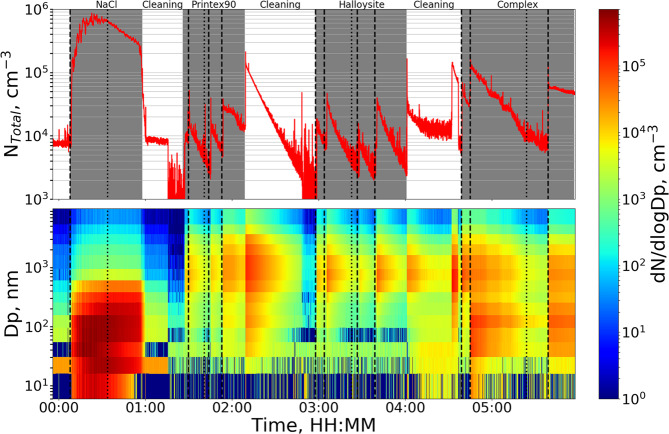
Total number concentration (top) and size distribution (bottom) time series plots measured by ELPI over the course of all aerosol experiments. Gray areas on the top plot indicate experiment periods, with the studied aerosol specified at the top of the plot, while the white areas indicate times where the chamber was flushed with clean air. Dotted vertical lines indicate collection of the impactor samples studied in detail, while the dashed vertical lines indicate particle production from the brush generator or atomizer.

An overview of the experimental process is provided by the time series plot of the size distributions and total number concentrations measured by ELPI throughout all aerosol experiments in Fig. 2. A similar plot of data from the SMPS is presented in Fig. 2 in the Supplementary Material.

### Size distribution comparison

A summary of findings from SEM analysis of each aerosol impactor sample is provided in Table 1, along with mean total number concentrations measured by the ELPI and SMPS.

**Table 1:** A table showing the number of images (N_img_), image resolution, total imaged area in um^2^, total number of recognized particles from both impactor stages for each of the sampled aerosols (N_p_) and the particle coverage on each grid, determined from total area covered by particles divided with the total imaged area (Coverage). Additionally the average total number concentrations measured by the SMPS and ELPI from 3 min before to 3 min after impactor collection are also reported (these were the same for all 2^nd^ and 3^rd^ stage samples of the same aerosol). No impaction spot was observed on the second stage of the pure NaCl sample, and therefore an image series of this stage was not acquired.

Aerosol	Stage, #	N_img_, #	Resolution, nm/px	Imaged Area, um^2^	N_p_, #	Coverage, µm_p_^2^/µm_img_^2^	N_SMPS_, cm^−3^	N_ELPI_, cm^−3^
NaCl	2	—	—	—	—	—	5.2 ∙ 10^5^	6.5 ∙ 10^5^
	3	25	7.3	4823	4594	0.020		
Halloysite	2	10	33.2	30854	241	0.016	3.1 ∙ 10^3^	3.8 ∙ 10^3^
	3	19	5.5	1630	3731	0.026		
Printex90	2	12	33.2	37025	210	0.031	3.8 ∙ 10^3^	4.7 ∙ 10^3^
	3	19	5.5	1630	3383	0.030		
Complex	2	11	57.1	34030	498	0.048	1.5 ∙ 10^4^	9.8 ∙ 10^3^
	3	12	7.3	2315	5797	0.033		

**Figure 3:**
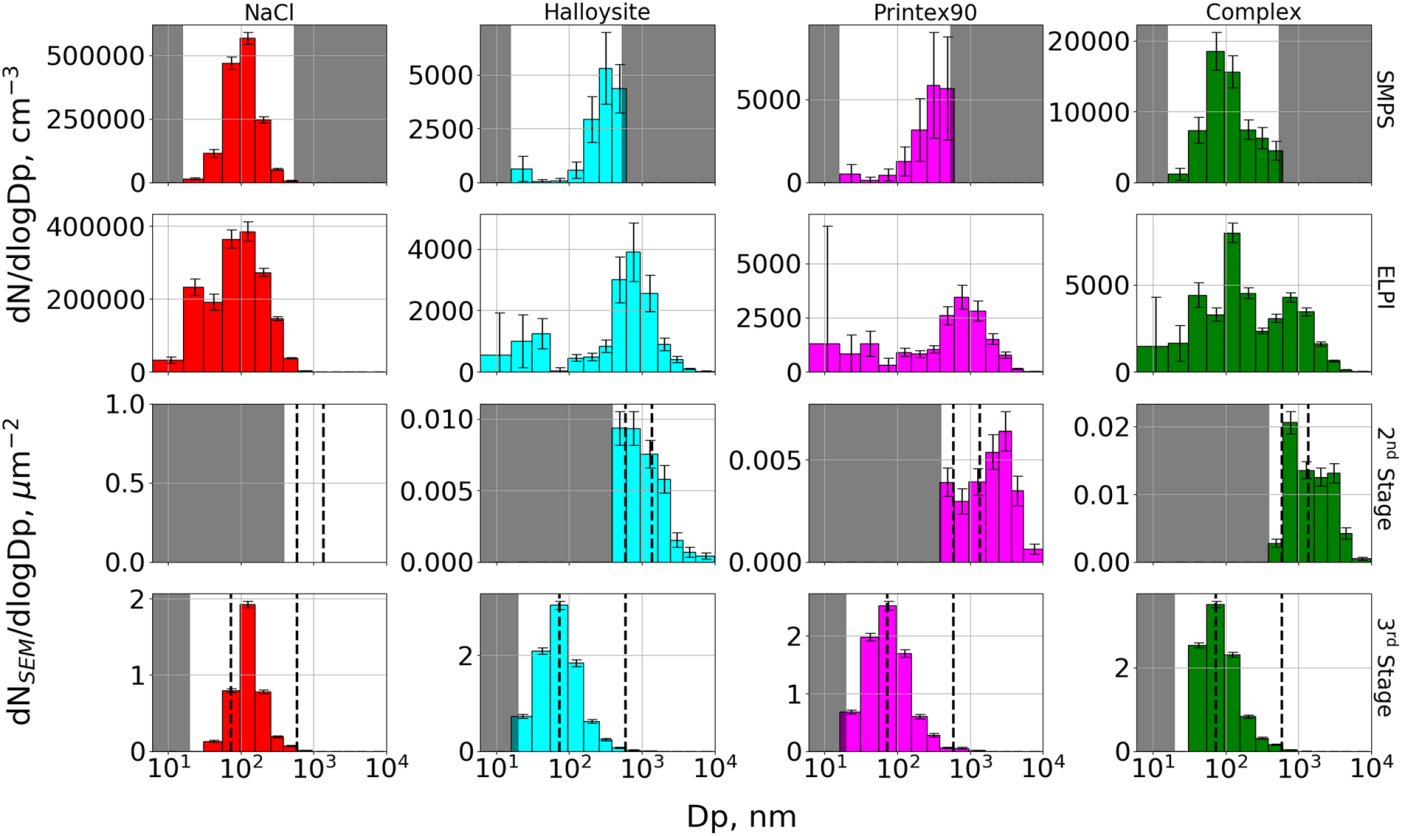
Particle size distributions of the four aerosols: NaCl (red), Halloysite (cyan), Printex90 (magenta), and complex (green), determined by SMPS (top row), ELPI (second row), and SEM analysis of the 2^nd^ (third row) and 3^rd^ (bottom row) MINI stage samples. Vertical dotted lines represent the relevant D_50_ cut-offs for the MINI stages, corresponding to 1.36, 0.59, or 0.073 µm. The 2^nd^ stage should therefore collect particles with sizes from 0.59–1.36 µm, while the 3^rd^ stage should collect particles from 0.073–0.59 µm. Grey-shaded areas are beyond the detection limit of the method, which for SMPS is from 16–533 nm, and for SEM/EDS was determined based on measurements of the clean TEM grid and Ni disc. It should be noted that the y-axis concentrations are not scaled between plots, and that SMPS and ELPI data are presented as number concentrations (cm^−3^), while SEM data is presented as number densities (µm^−2^) with uncertainties determined from counting statistics as √N. SMPS and ELPI uncertainties are determined from standard deviations of the averaged runs, covering the period from 3 minutes before to 3 minutes after impactor collection. Finally, it should be noted that the diameters from each method may not be directly comparable as the SMPS reports electrical mobility diameters, the ELPI reports aerodynamic diameters, while the SEM results are given in equivalent circular diameters.

Typical SE images before and after segmentation from both stages of the four aerosol samples are shown in Figs. 3 and 4 in the Supplementary Material. The recognized particles were binned according to their D_eq_, using the size bins of the ELPI. Particle number densities for each size bin and each sample were determined by normalizing particle counts with the total imaged area. This allows comparison between number densities found on the same stage for different samples. However, a comparison between the two stages cannot be made directly, as the particle sizes and impact area on the 2^nd^ stage are significantly larger than those of the 3^rd^ stage, giving little overlap in the PSD as shown in Fig. 3. Finally, the particle count in each bin was divided by the ELPI bin width to give number densities in the form dN_SEM_/dlogDp.

**Figure 4:**
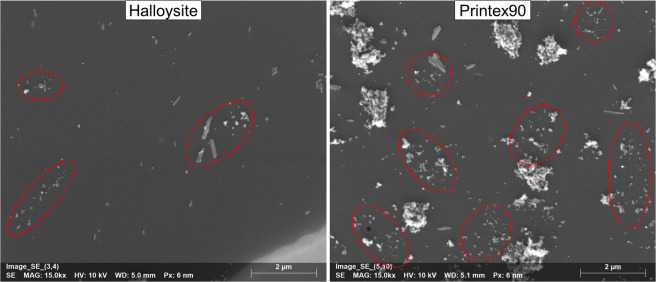
SE images showing examples of inhomogeneous particle densities on the 3^rd^ stage of the Halloysite (left) and Printex90 (right) impactor samples. Areas with number densities significantly higher than the surroundings have been circled in red dashed lines.

For comparison with SMPS and ELPI measurements the average size distribution was determined from scans covering 3 min before to 3 min after impactor sampling. To ease comparison between all size distributions, results were converted to the same size scale. Therefore, the SMPS size distributions were resized using the ELPI size bins, before converting to dN/dlogDp (the data before conversion is shown in Fig. 5 in the Supplementary Material). The ELPI binned PSDs for each aerosol obtained by SMPS, ELPI, and from SEM analysis of the 2^nd^ and 3^rd^ MINI stages are presented in Fig. 3. It should however be noted, that the equivalent diameters reported by the three methods may not be directly comparable, and could lead to some of the observed biases.

**Figure 5:**
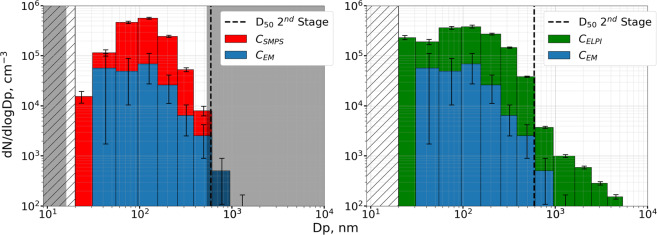
Airborne number concentrations estimated using Eq. (1) from EM data of the 3^rd^ impactor stage of the NaCl experiment (blue), compared to airborne concentrations measured by SMPS (left in red) and ELPI (right in green). Error bars on the EM data include counting statistics, the uncertainty of the *A*_*eff*_ parameter, and uncertainties of the C_eff_ expression estimated by altering D_50_ according to previously determined uncertainties (73 ± 8 nm). The uncertainties of the SMPS and ELPI data are represented as the standard deviation of the averaged SMPS or ELPI scans. Dark grey areas are outside the SMPS size detection limits, while the hashed area represents particles too small to be recognized during EM analysis. It should be noted that the diameters from each method may not be directly comparable as the SMPS reports electrical mobility diameters, the ELPI reports aerodynamic diameters, while the SEM results are given in equivalent circular diameters.

NaCl particles are compact cubes, meaning that the equivalent diameters reported by the ELPI and SMPS should be in good agreement with SEM results. This fits well with our observations, where all instruments found one mode centered at the 96–156 nm bin. The reported sizes are consistent with the approximately 100 nm sizes expected from atomizing a solution of NaCl^55^. The modes determined by SMPS and ELPI are slightly broader towards the smaller sizes compared to the SEM mode, but this is expected as the mode spans below the 3^rd^ stage D_50_ at 73 nm, from where a decreasing particle fraction is collected. No particles were observed on the 2^nd^ impactor stage, which primarily collects particles larger than 590 nm, consistent with the SMPS and ELPI results.

For the Halloysite aerosol, the SMPS shows a main mode near its upper detection limit at 256–382 nm as well as a much smaller mode near its lower detection limit. Here it should be mentioned that the SMPS size range stops at 532.8 nm. An artificial number reduction can therefore occur in the 382–604 nm bin, which is only partially within the SMPS size range, compared to the fully resolved 256–382 nm bin. Taking this into account, the main SMPS mode is consistent with the ELPI measurements. The ELPI covers a broader size range, showing a mode centered at the 604–949 nm size bin. The ELPI also detects an additional smaller mode at the 31–55 nm bin. This smaller ELPI mode could be linked to the SMPS mode at the lower detection limit, with the size offset resulting from differences in their reported equivalent diameters. An overall good agreement is found between the SMPS and ELPI PSDs despite anticipated discrepancies, as the electrical mobility of fibrous particles is influenced by their length whereas the aerodynamic behavior is more associated with their width^46^. The agreement between the methods could result from fibers gathering in bundles with shapes close to that of large spheres. This was supported when investigating the 2^nd^ stage impactor sample, shown in Fig. 3 in the Supplementary Material. The PSD from the SEM analysis of the 2^nd^ Halloysite stage showed one mode, peaking across two bins from 382–949 nm, which fits very well with the SMPS and ELPI results. However, the 3^rd^ stage PSD shows a single mode peaking at the 55–96 nm bin, which is at a concentration minimum of the SMPS and ELPI distributions.

The Printex90 aerosol shows a PSD almost identical to that of the Halloysite aerosol for the SMPS and ELPI. Again the SMPS shows a mode not fully resolved at its upper size limit as well as an indication of a smaller mode at its lower size limit. The highest ELPI concentration is at the 604–949 nm size bin, with a smaller mode at the 31–55 nm bin. Since the primary particle size of the Printex90 powder is approximately 14 nm, the 604–949 nm particles must be agglomerates consisting of thousands of primary spheres. Agglomerates have been reported in previous studies of Printex90, but at significantly smaller sizes of 30–200 nm^56^, though different aerosolizing techniques were applied. It is possible that the brush generator acts as a soft aerosolizing method, producing very large and loosely agglomerated particles. The SEM analysis of the 2^nd^ Printex90 stage shows the highest number density at the 2470–3660 nm bin, which is much larger than the ELPI peak concentration. However, when inspecting the SEM images, exemplified in Figs. 3 and 4 in the Supplementary Material, it is seen that many of the detected particles consist of overlapping micrometer-sized particles. These could have been airborne as individual particles, and have formed via co-deposition. The 3^rd^ stage of the Printex90 sample shows a single mode, almost identical to that of the Halloysite 3^rd^ stage, with the peak density at the 55–96 nm bin. Again this is in poor agreement with the size distributions measured by SMPS and ELPI, which both display concentration minima in the 55–96 bin.

The complex aerosol should ideally be a mixture of the above, resembling a combination of all three size distributions, assuming no agglomeration while airborne. The complex SMPS PSD displays a single mode at the 55–96 nm bin, which is slightly below the mode observed for the pure NaCl aerosol. Sizes larger than the 55–96 nm mode show an elevated number concentration compared to the pure NaCl aerosol. This indicates the presence of larger particles, consistent with the pure Halloysite and Printex90 aerosols. The broader size range of the ELPI shows three modes centered at 31–55, 96–156 and 604–949 nm. The mode near 100 nm is consistent with the mode observed for the pure NaCl aerosol, while the 31–55 and 604–949 nm modes fit well with the pure Halloysite and Printex90 measurements. As such, the complex aerosol measured by SMPS and ELPI resembles a mixture of the three primary size distributions, but without prior knowledge it would not be possible to distinguish between particle types. The 2^nd^ stage of the complex aerosol shows a peak concentration at the 604–949 nm bin, which fits well with the ELPI observations. The 3^rd^ stage size distribution displays a single mode at 55–96 nm, similar to the 3^rd^ stage Printex90 and Halloysite samples. This fits well with the SMPS measurements, while it is in between the two lowest ELPI modes. Intriguingly, the correlation between the ELPI, SMPS, and SEM size distributions is better for the complex aerosol compared to the measurements of the pure Printex90 and Halloysite aerosols.

It should be noted that some of the TEM grid squares broke upon particle impaction, with a total of 3, 1, 4, and 4 broken squares for the NaCl, Halloysite, Printex90, and complex samples respectively (see overview images in Fig. 6 in the Supplementary Material). It was therefore not possible to pass the line of images needed for analysis directly through the impact center for all of the imaged areas^36^. This is also seen in Fig. 6 in the Supplementary Material, where an estimated orifice position and the imaged areas are indicated. This could reduce the representation of particles near the upper collection limit for the 3^rd^ stage (590 nm), as these typically impact near the center of impaction^57^. However, as this would not produce any particles in the 55–96 nm range, it does not explain the poor correlation between the 3^rd^ stage size distribution and those determined by SMPS and ELPI.

**Figure 6:**
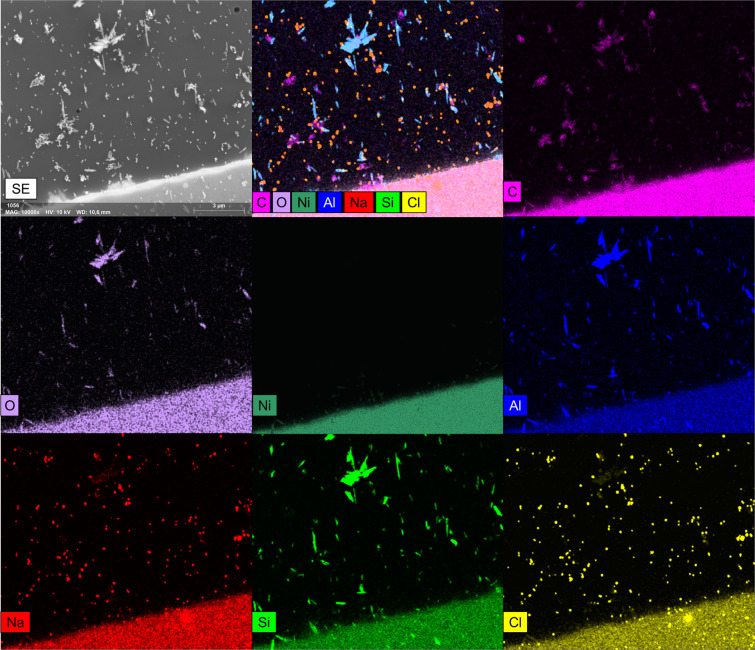
Top left: A Secondary electron image of an area of the complex aerosol sample from the 3^rd^ stage. Top middle: An overlay of all elemental maps of the secondary electron image. Individual element maps of C, O, Ni, Al, Na, Si, and Cl are shown in the remaining images, with the given element marked in the left bottom corner of each map. Maps were generated by summing pixel EDS spectra in 4 × 4 areas and converting the X-ray count in each element energy region to a normalized pixel intensity of a given color. The online deconvolution setting in the ESPRIT software was used to correct for possible peak overlaps.

To further investigate the large discrepancies between the real time and SEM measurements, the images from the pure Printex90 and Halloysite aerosols were carefully reviewed. Here it was noted, that the deposition patterns on the 3^rd^ stage were not homogeneous. Instead they displayed several small areas with dense particle populations, as marked by red circles in Fig. 4. This pattern was especially prominent near the center of impaction, and was most obvious on the Printex90 sample. These patterns strongly indicate that larger particles impacted onto the substrate and either shattered upon impaction or bounced off the substrate, leaving smaller residual particles behind, as has also been reported in the literature^58–62^. The same process may have occurred on the 2^nd^ impactor stage, but here it was not visible due to the rough surface of the Ni disc. It would however have decreased the number of large particles and significantly increased the number of smaller particles, which could be transported onwards to the 3^rd^ stage. This offers a viable explanation for the large discrepancies between the real time and SEM size distributions.

The pure NaCl sample did not display the same impact patterns, indicating that the NaCl aggregates/crystals did not break upon impact. Aerosol samples collected via impaction should therefore be carefully inspected for non-homogeneous deposition patterns, i.e. small areas with dense particle population. Such areas could indicate agglomerate fracturing, artificially increasing the number density of smaller compared to larger particles. Ideally, impaction should not be used to collect aerosols containing large agglomerates, where alternative and softer sampling methods are needed instead, e.g. filtration^63^, electrostatic sampling^64,65^, or thermophoretic sampling^66–68^. However, impaction is well suited for sampling homogenous, stable, and non-agglomerated materials.

A vital step in utilizing SEM/EDS for exposure assessments lies in linking the sample observations to the properties of the original aerosol, as it is the exposure to airborne particles which is of interest. In previous work^36^, an expression was proposed to link airborne concentrations (*C*_*EM,Dp*_, cm^−3^) to the number density of particles observed on samples from the 3^rd^ impactor stage (*N*_*imp,Dp*_, µm^−2^). To do so, it is necessary to consider the impactor collection efficiency at each particle size (*C*_*eff,Dp*_, unitless) and the total volume of air that passed through the impactor during sampling. The latter is calculated from the flow through the impactor (*Q*, cm^3^/s) and the sampling time (*t*, s). It is furthermore necessary to account for the influence of several complex contributions, including the effective particle collection area, particle wall loss, and particle bounce. The effect of each of these contributions are difficult to measure and may depend on particle characteristics e.g. size, type, and physical state as well as impactor characteristics e.g. design, wall material/roughness, and impaction surface. In previous work, a simple approach was taken, where a parameter (*A*_*eff*_) was estimated from calibration experiments with polystyrene latex beads (PSL). Here an *A*_*eff*_ value of (1.12 ± 0.60)∙10^6^ µm^2^ was found, and airborne concentrations were given by:

1 $${C}_{EM,Dp}=\frac{{A}_{eff}}{Q\ast t\ast {C}_{eff,Dp}}\,\ast \,{N}_{imp,Dp}$$

In this work the expression is tested by estimating number concentrations for each size bin of the NaCl sample, which is compared to SMPS and ELPI results in Fig. 5. Due to the impaction artefacts observed on the Halloysite, Printex90, and complex samples, the expression could not be used for those data.

It is seen that the shape of the NaCl PSDs are similar for the EM, SMPS, and ELPI data. This indicates that the impactor SEM/EDS analysis can reproduce airborne size distributions. However, the airborne number concentrations estimated from Eq. (1), are factors of 8–9 lower than the SMPS results, and factors of 5–10 lower than the ELPI data, if sizes near and above the 2^nd^ stage D_50_ are excluded. The *A*_*eff*_ factor is therefore apparently too low for the NaCl experiment. There are several possible explanations for the observed discrepancies. The most probable explanation is the installation of impaction plates in the 1^st^ and 2^nd^ impactor stages, which were not installed when the *A*_*eff*_ parameter was determined. The installation of the two additional impactor stages alters the flow through the impactor and could increase diffusion or wall losses, thus requiring a higher *A*_*eff*_ parameter to correct. Many previous studies have investigated bounce and losses in impactors, but never in relation to microscopy analysis. It is therefore clear that the impactor can for now only be used to get an estimate of the total particle number concentrations within a factor of 10. However, additional work on calibration with multiple stages and an improved understanding of the impactor will increase precision of particle number measurements. Considering that the ELPI and SMPS are measuring with differences of up to a factor of 5 on the complex aerosol, the impaction method appear to enable an independent measure with far more additional information than the two standard methods.

### Elemental particle classification

To demonstrate measurements of elemental composition by SEM/EDS analysis, the 2^nd^ and 3^rd^ stage samples of the complex aerosol were analyzed by EDS mapping. Maps can display the distribution of elements within the imaged area as EDS spectra are available for each pixel. To increase the counting statistics, individual pixel spectra are summed in 4 × 4 pixel areas, which decrease the map resolution, but gives a higher map quality with more reliable counts. Examples of maps from the 3^rd^ impactor stage are shown in Fig. 6. A similar figure for the 2^nd^ stage is shown in Fig. 7 in the Supplementary Material.

**Figure 7:**
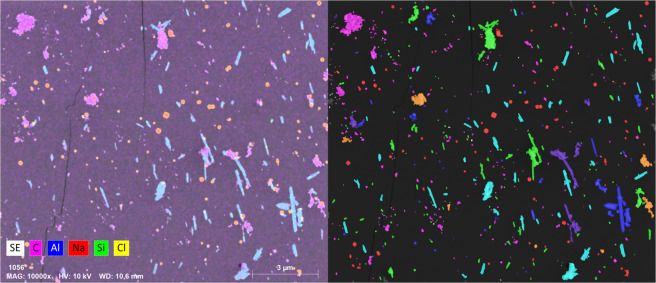
A SE image overlaid with relevant EDS maps (left) from the complex aerosol collected at the 3^rd^ impactor stage, along with the classified particle image (right). Classification was made with the scheme presented in Table 2. Color codes of the classified image are stated in Table 2.

As seen from Fig. 6, mapping can be used to visualize the spatial distribution of elements in a sample, distinguishing particles of different composition, even without any prior knowledge of the sampled aerosol. Clear orange NaCl particles are observed from the overlaying yellow Cl and red Na maps, cyan colored Halloysite fibers from the overlaying blue Al and green Si maps, and magenta Printex90 particles from the C map. Maps can therefore identify primary particle types and give information on the mixing state of agglomerates, as seen on the overlay of all maps at the top center image. It is however necessary to consider co-deposition, which can artificially increase the number of agglomerates when particles deposit on top of each other. This can have a major influence both on the aerosol mixing state, but also on the aerosol PSD as shown by Kandler *et al*.^34^. They recommend sample coverages of 0.03–0.05, which are similar to those in this study (see Table 1). When fulfilling these requirements, the method can differentiate primary particle types and agglomerates in an unknown aerosol sample, and give indications of the PM sources by distinguishing e.g. soot, metal particles, minerals, and salts. The method can thus bring relevant information for PM exposure, risk assessments, and pollution abatement strategies^69–72^.

Maps, such as the one presented in Fig. 6, must be interpreted with care. For instance, all elements show an elevated number of X-ray counts on the bulk Ni grid at the bottom of the image. This increase is not related to the presence of the elements, but simply from the elevated number of X-rays in the spectrum background, due to a stronger interaction between the electron beam and bulk Ni grid. Normally this is corrected by peak fitting algorithms and background subtraction procedures, but these are highly uncertain due to the low X-ray count in each 4 × 4 pixel area. The same phenomenon may occur for large bulk particles, which can display traces of elements that are not actually present. This uncertainty is reduced, when the composition of individual particles are investigated, as the X-ray counts from particles consisting of hundreds to thousands of pixels are significantly higher than the 4 × 4 pixel areas of the map, thus allowing higher quality background corrections. For cases with non-beam sensitive particles, the acquisition time can be increased, allowing better counting statistics and more reliable maps, albeit at the cost of longer analysis time.

To identify which particle types dominate the different PSD size ranges, it is necessary to distinguish particles, using a classification scheme. Since the primary particle types can be visualized directly from the EDS maps, the relevant classes are already known. Additionally, the mixing state of all particles is visible from the EDS map, and the classification scheme can therefore be setup and perfected by iteratively altering the composition criteria to ensure that particles fall into distinct categories. This procedure was used to setup a classification scheme that differentiate between the particle types of the complex sample. Particles were divided into eight different classes, depending on their individual elemental composition. Since Si was specific to Halloysite fibers, while Na and Cl were specific for NaCl particles, it was found that thresholds of ≥1 at% were optimal to distinguish these classes. A much higher criterion of ≥60 at% was needed for the Printex90 class, as all particles displayed significant carbon content due to the TEM grid substrate. Additional classes were set up to identify agglomerates of the primary particle types, using combinations of the primary class criteria. Oxygen is not included as it is present in many different compounds and substrate, and would not help to distinguish different particle classes. The eight classes are listed in Table 2, along with their respective elemental concentration criteria.

**Table 2:** Elemental composition criteria of the particle classification scheme used to divide individual particles into classes of primary or agglomerated particles. The class “All” refers to agglomerates containing all three compounds (“NaCl+Halloysite+Printex90”). The colors listed in the last column correspond to those used in the classified particle image in Fig. 7.

Classes	C, at%	Si, at%	Na, at%	Cl, at%	Color
NaCl	—	—	≥1%	≥1%	Red
Halloysite	—	≥1%	—	—	Cyan
Printex90	≥60%	—	—	—	Magenta
NaCl + Printex90	≥60%	—	≥1%	≥1%	Orange
NaCl + Halloysite	—	≥1%	≥1%	≥1%	Blue
Halloysite + Printex90	≥60%	≥1%	—	—	Green
All	≥60%	≥1%	≥1%	≥1%	Purple
Unclassified	<60%	<1%	<1%	<1%	White

An example of a SE image from the 3^rd^ impactor stage of the complex aerosol sample overlaid with relevant elemental maps are shown in Fig. 7, along with the corresponding classified image made with the scheme and color codes presented in Table 2. The original SE image is displayed in Fig. 8 in the Supplementary Material.

**Figure 8:**
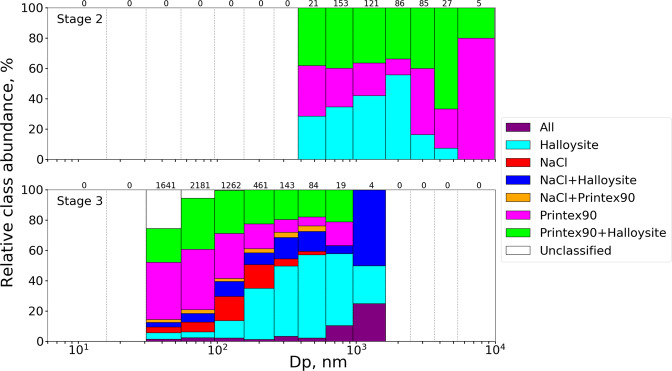
The relative abundance of particle classes in each size bin of the size distributions found on the 2^nd^ (top) and 3^rd^ (bottom) stage of the complex aerosol sample. The number of particles in each bin is written above the size bin, which corresponds to the numbers of the complex sample in Fig. 3. The All class refers to agglomerates consisting of NaCl, Halloysite, and Printex90 particles.

From Fig. 7 it is seen that the classification scheme presented in Table 2 is able to distinguish primary particles and agglomerates, as well as separate agglomerates into relevant classes consisting of combinations of the primary particles. It can however be challenging to produce a classification scheme, which functions at all particle sizes. Especially small sizes, with limited X-ray counts, can present an overestimated number of carbon based particles, as the substrate contributes substantially but highly varying to the overall X-ray count. This could be improved by increasing the pixel dwelltime, which would increase the X-ray count and lower the uncertainty from counting statistics, but would also increase the analysis time and the risk of beam damage. One might run a fast map for beam sensitive elements such as Cl in NaCl and then a longer one for elements that can withstand irradiation. Additionally, it can be challenging to define when a particle is an agglomerate^34^. For example, if a 70 nm NaCl particle coagulates with a 1 µm Printex90 particle, should it still be considered an agglomerate, despite limited contribution from the NaCl particle to the overall physicochemical properties. Still, it is possible to generate a classification scheme, based on the EDS map without any prior knowledge of the sample, which can distinguish between most primary particle classes and their agglomerates. This provides information on the mixing state of the entire aerosol, and allows for class specific size and shape information, which can be presented as class separated size distributions as shown in Fig. 9 in the Supplementary Material. Alternatively, the aerosol mixing state can be displayed as the relative abundance of each class in the overall PSD, as exemplified in Fig. 8.

**Figure 9:**
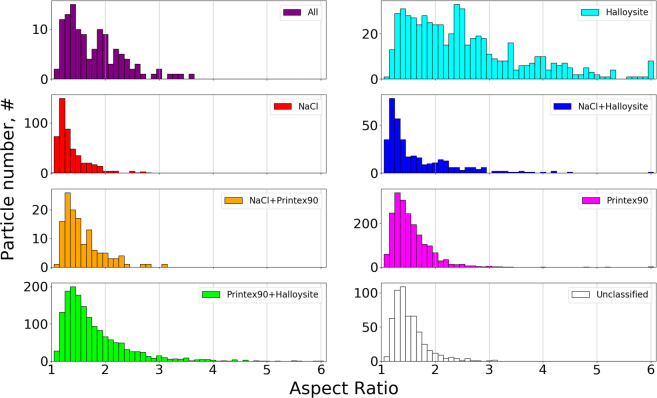
Aspect ratio distributions for each particle class detected on the 3^rd^ stage of the complex aerosol sample. Particles were divided into AR bins with a width of 0.1. Particle numbers on the y-axis include all particles in the given class detected across all 12 images.

Figure 8 shows that approximately 40% of all particle sizes on the 2^nd^ impactor stage consist of Halloysite and Printex90 agglomerates, while the primary Halloysite fibers are almost exclusively seen below 2 µm. Printex90 particles are observed at all particle sizes, but has the highest relative abundance at the larger sizes above 2 µm. These observations are consistent with the 2^nd^ stage pure aerosol samples, where the Printex90 mode was found at much larger sizes (2470–3660 nm) than the pure Halloysite mode (382–949 nm), seen in Fig. 3. Additionally, a complete absence of NaCl is observed on the 2^nd^ stage, consistent with their smaller sizes, though agglomerates of NaCl could still occur. This could be explained from a limited X-ray contribution of Na and Cl when agglomerating with micrometer sized Halloysite or Printex90 particles.

For the 3^rd^ stage, 20–30% of all particle sizes consist of Halloysite and Printex90 agglomerates, while pure Printex90 particles dominate sizes below 100 nm, making up almost 40% of the analyzed particles. This is consistent with the 3^rd^ stage pure Printex90 size distribution observed in Fig. 3, where the mode is located at 55–96 nm. Some of the small particles classified as Printex90 could also be misclassifications due to the higher substrate contribution. The relative abundance of Printex90 particles decreases with size to a minor constituent above 200 nm. The NaCl particle class is only found at sizes below 500 nm, with the highest abundance observed from 100–200 nm. The individual size distribution of the NaCl class, seen in Fig. 9 in the Supplementary Material, shows that the mode is located at 96–156 nm, consistent with the NaCl PSD in Fig. 3. The NaCl+Halloysite class is a minor constituent at all sizes of the aerosol, accounting for 5–10%. The Halloysite particles become the most abundant above approximately 200 nm, reaching 30–50% of the overall particle number. Note that the number of particles also drop with increasing size, so when examining the pure Halloysite size distribution presented in Fig. 9 in the Supplementary Material, it is seen that the classified Halloysite mode is located from 156–256 nm. This is at a substantially larger size than the Halloysite PSD in Fig. 3, where the mode is located at 55–96 nm. Finally, a fraction of unclassified particles are also observed at sizes below 100 nm, most likely due to the low X-ray yield of the small particles, making it difficult to give accurate composition measurements. Future research should verify the aerosol mixing state reported by SEM/EDS analysis through comparison to alternative measurement techniques e.g. aerosol mass spectrometry.

The SEM/EDS analysis also provides morphological information on each individual particle, which can be combined with the elemental class information. This makes it possible to generate aspect ratio (AR) shape distributions for each class in the aerosol, as shown in Fig. 9.

From Fig. 9 it is seen that the AR distribution of NaCl is very narrow and centered close to 1, with only a few particles presenting ratios above 1.5. This fits very well with the dense and cubic shapes expected for NaCl particles. The Printex90 AR distribution is centered at 1.3–1.4, which is less square than the NaCl. Additionally the distribution is much broader displaying a significant number of particles with AR as high as 2. This indicates that the Printex90 class contains a significant number of agglomerates, since the primary spheres should have AR values close to 1. The Halloysites show a much broader AR distribution, ranging from 1.2 to 6, which reflects their fibrous shape and is consistent with AR values reported previously^44,45^. All agglomerate classes involving Halloysites (All, Printex90 + Halloysite, and NaCl + Halloysite) display relatively broad AR distributions, typically extending up to 3 or 4, whereas the other classes (NaCl, Printex90, NaCl + Printex90, and Unclassified) rarely show AR higher than 2. It is therefore clearly seen that the particle shape of the Halloysite class differs significantly from the two other primary particles, and that this shape is descriptive for all agglomerates involving Halloysites. Shape distributions can therefore be an important tool for distinguishing fibers from other particle shapes, even without any prior knowledge of the sample. This can be crucial in risk assessment of particle aerosols, as the fibrous shape and high AR are associated with adverse health effects^42,45^.

## Conclusion

Four aerosols consisting of NaCl, Halloysites, Printex90, or a mixture of the three (complex) were measured by SMPS and ELPI, and characterized by SEM/EDS analysis of impactor collected samples. The PSDs derived by SMPS and ELPI were in good agreement for all four aerosols (Fig. 3), despite expected discrepancies for the fibrous particles. It is possible that the brush generator used for aerosolizing the powders, produced large and near spherical agglomerates of the smaller primary particles, resulting in similar shapes and similar size distributions for the two particle types. This was supported by observations of large µm sized agglomerates on the 2^nd^ impactor stage. The SMPS and ELPI measurements were consistent with the impactor-SEM size distributions for the NaCl and complex aerosol samples. However, large discrepancies were observed for the Halloysite and Printex90 aerosols (Fig. 3). This was caused by agglomerates, which shattered or bounced upon impaction, leaving behind small residue particles (Fig. 4). As a result, the number densities of small particles were overestimated relative to larger ones. It was therefore concluded that impactor samples should be scanned for such deposition patterns, in which case alternative and softer collection methods should be used.

Airborne number concentrations were estimated, based on number densities of the NaCl impactor sample, using a previously and experimentally derived expression. These were compared to SMPS and ELPI measurements, showing good agreement for the PSD shapes, but airborne concentrations were underestimated by a factor of 5–10 (Fig. 5). This was probably due to discrepant conditions during derivation of the expression and the present impactor collection. Additional work is needed on calibration of the impactor, to enable more precise and reliable particle number estimates, which combined with the highly detailed characterization by SEM/EDS, can supply many of the relevant parameters for exposure and risk assessments of particles.

It was shown that SEM/EDS maps can visualize the spatial distribution of elements within the entire analyzed area, allowing quick and direct identification of primary particle types and agglomerates without prior knowledge of the sample (Fig. 6). This insight was used to develop and implement a classification scheme, dividing individual particles into relevant classes based on their elemental composition (Table 2). The classification scheme was optimized by iterative alteration of the class criteria and comparison between the classified image and elemental maps (Fig. 7). It was shown that the classification performed well for larger sizes. Some discrepancies were observed for small particles with limited X-ray counts. This could be improved by increasing the map dwelltime, but would also increase the analysis time and risk of beam damage. The particle classification enabled plotting of the relative abundance of each class in the overall PSD (Fig. 8), which describes the aerosol mixing state. The observations matched well with measurements of the individual NaCl, Halloysite, and Printex90 aerosols, showing that the SEM/EDS analysis is capable of differentiating between particle types in complex aerosols. The classification also allowed class separated size and shape analysis (shown in Fig. 9 in the Supplementary Material and in Fig. 9). A class specific aspect ratio distribution was demonstrated, where the fibrous nature of the Halloysite particles was easily distinguished, along with the dense cubic structure of the NaCl particles.

Overall, it was shown that SEM/EDS analysis of impactor collected samples can provide a detailed characterization of aerosols, beyond those achievable by SMPS and ELPI. The analysis can provide sufficient particle data for statistical analysis of a sampled aerosol population within 1–2 hours per sample, and the acquisition process can be automated to minimize user intervention except for the initial setup. The analysis output includes particle size, shape, mixing state, and elemental composition for each individual particle. Thus, particles can be classified based on their physicochemical properties, distinguishing primary particles and agglomerates in complex aerosols. This also permits class specific analyses, which is particularly relevant in exposure scenarios with multiple PM sources, where source apportionment is needed e.g. during ambient conditions^70–72^ or where a complex aerosol is generated directly from a single source such as during welding. Additionally, identification of specific particles can be targeted, e.g. based on iron content or fibrous structure^12,42^. However, further research is needed to improve the link between SEM/EDS sample observations and the original aerosol and to verify the SEM/EDS reported aerosol mixing state by comparison to other measurements techniques. Alternative sampling methods should also be investigated for sampling of aerosols with weakly bonded agglomerates, as these are not well suited for collection by impaction. SEM/EDS analysis can bring crucial knowledge on particle properties of high relevance for PM risk assessments, exposure assessments, epidemiological studies, and the development of preventive strategies for PM pollution.

## Supplementary information

Supplementary Material
