# Supplementary material for: Complex Aerosol Characterization by Scanning Electron Microscopy Coupled with Energy Dispersive X-ray Spectroscopy
Source: Sci Rep. 2020 Jun 4;10:9150. doi: 10.1038/s41598-020-65383-5 (PMC7272469; doi:10.1038/s41598-020-65383-5)
Supplement: Supplementary file 1 — Supplementary Material [file 41598_2020_65383_MOESM1_ESM.docx]

# Supplementary Material for:

# Complex Aerosol Characterization by Scanning Electron Microscopy Coupled with Energy Dispersive X-ray Spectroscopy

Anders Brostrøm^1,2^, Kirsten I. Kling^1^, Karin S. Hougaard^2^, and Kristian Mølhave^1*^

^1^Technical University of Denmark, DTU Nanolab – National Centre for Nano Fabrication and Characterization, Fysikvej, Building 307, 2800 Kgs Lyngby, Denmark

^2^National Research Centre for the Working Environment, Lersø Parkallé 105, 2100 Copenhagen, Denmark

*Corresponding author: krmo@dtu.dk

## Images of a clean TEM grid and Ni disc

A clean TEM grid and a clean Ni disc were also investigated by SEM to ensure that no particles were detected, see SI Figure 1. The images bear no indications of particles on the clean TEM grids. However, the rough surface of the Ni disc could be misinterpreted as particles, but the surface roughness typically displayed as areas with poor contrast and D_eq_ < 400 nm. Particles generally present with much higher pixel intensities, and a global threshold could therefore be used to distinguish particles from the surface of the Ni disc, if segmented areas with D_eq_ < 400 nm were excluded from the analyzes. As the 2^nd^ impactor stage primarily collects particles larger than the 590 nm cut-off, this criterion should not have a significant effect on the results. For the 3^rd^ impactor stage however, which primarily collects particles below 400 nm, the Ni disc could not be used as the surface roughness would obscure the particles. It is therefore important to use a TEM grid on the 3^rd^ impactor stage.


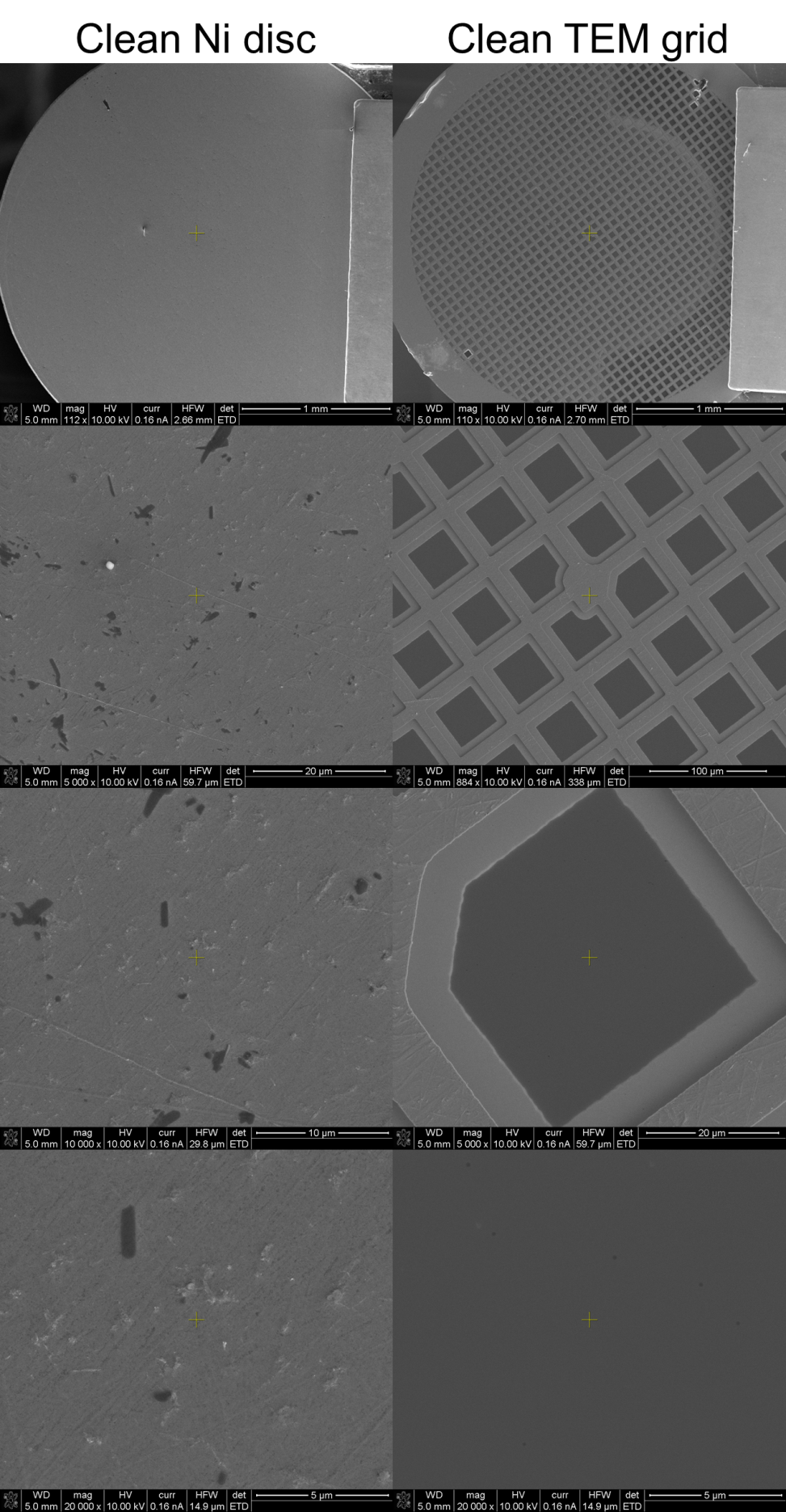


SI Figure 1. Overview and up to 20k magnification images of a clean Ni disc and TEM grid.

## Time Series SMPS


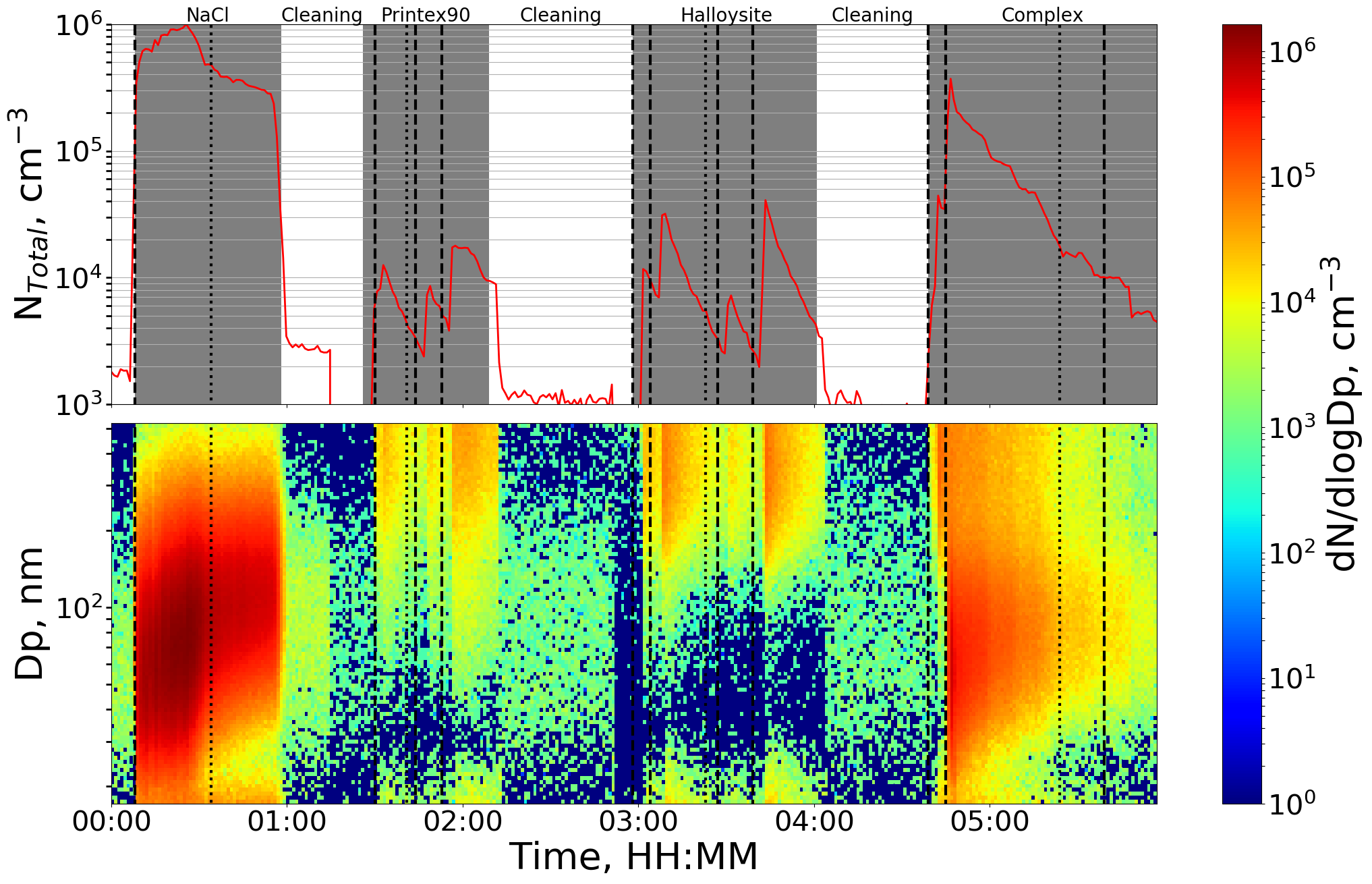


SI Figure 2. SMPS total number concentration and size distribution time series plots, over the course of all aerosol experiments. Gray areas on the top plot indicate an experiment, with the used aerosol specified at the top of the plot, while the white areas indicate times where the chamber was flushed. Vertical dotted lines indicate times where the impactor samples analyzed in detail were collected, while vertical dashed lines indicate times where the atomizer or brush generator was active to release a burst of particles into the chamber. It should be noted that the SMPS was disconnected in the cleaning periods.

## Size Distributions – SMPS and ELPI


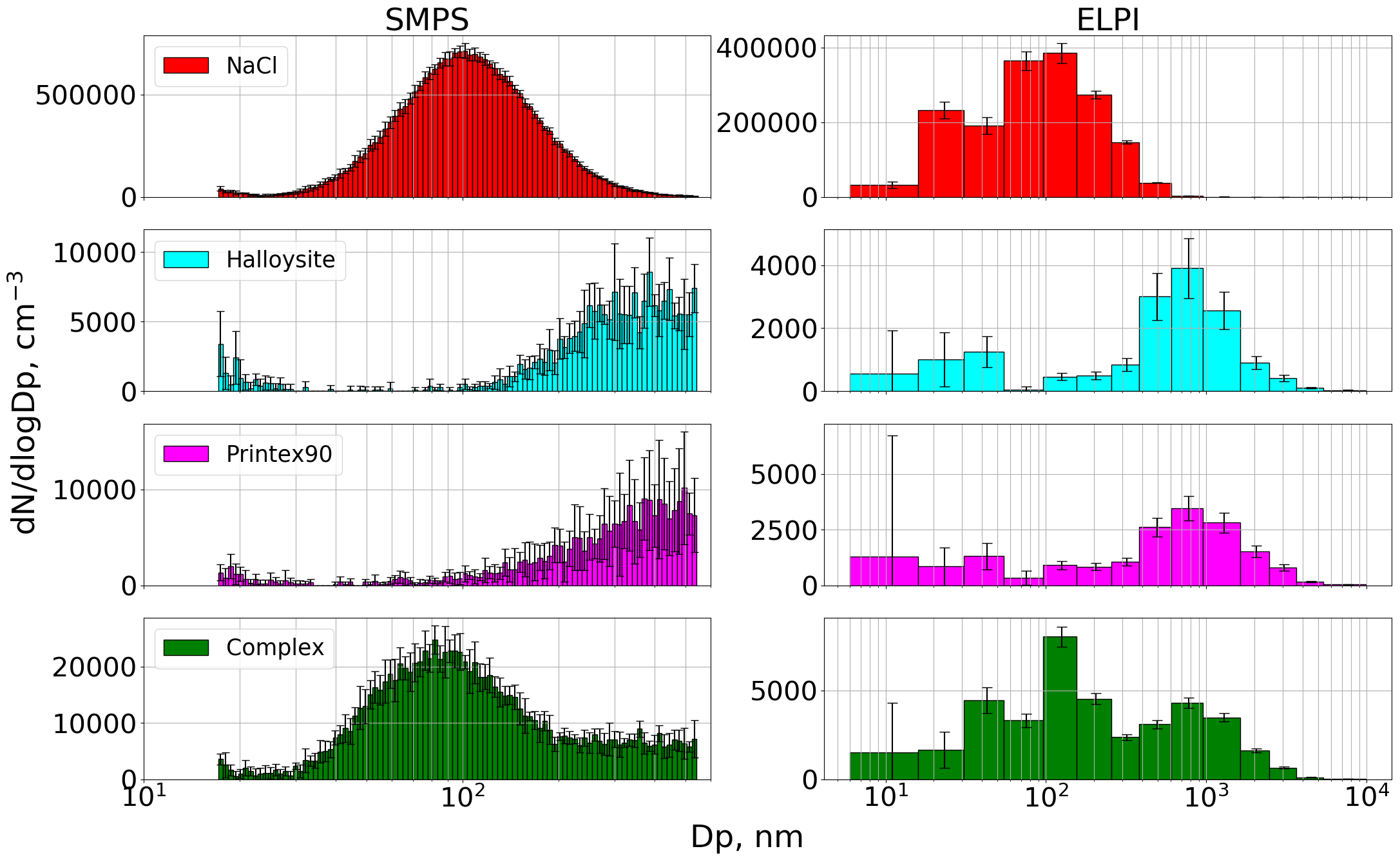


SI Figure 3. Particle size distributions measured by SMPS (left) and ELPI (right) of the four aerosols: NaCl (red), Halloysites (cyan), Printex90 (magenta), and the complex aerosol (green). Particle size distributions are determined as averages of scans from 3 minutes before to 3 minutes after the dotted lines marked in Figure 2, with error bars representing standard deviations within this timeframe. It should be noted that the y-axes are not equally scaled, and that size ranges differ between ELPI and SMPS.

## Overview images of the four aerosol samples

##
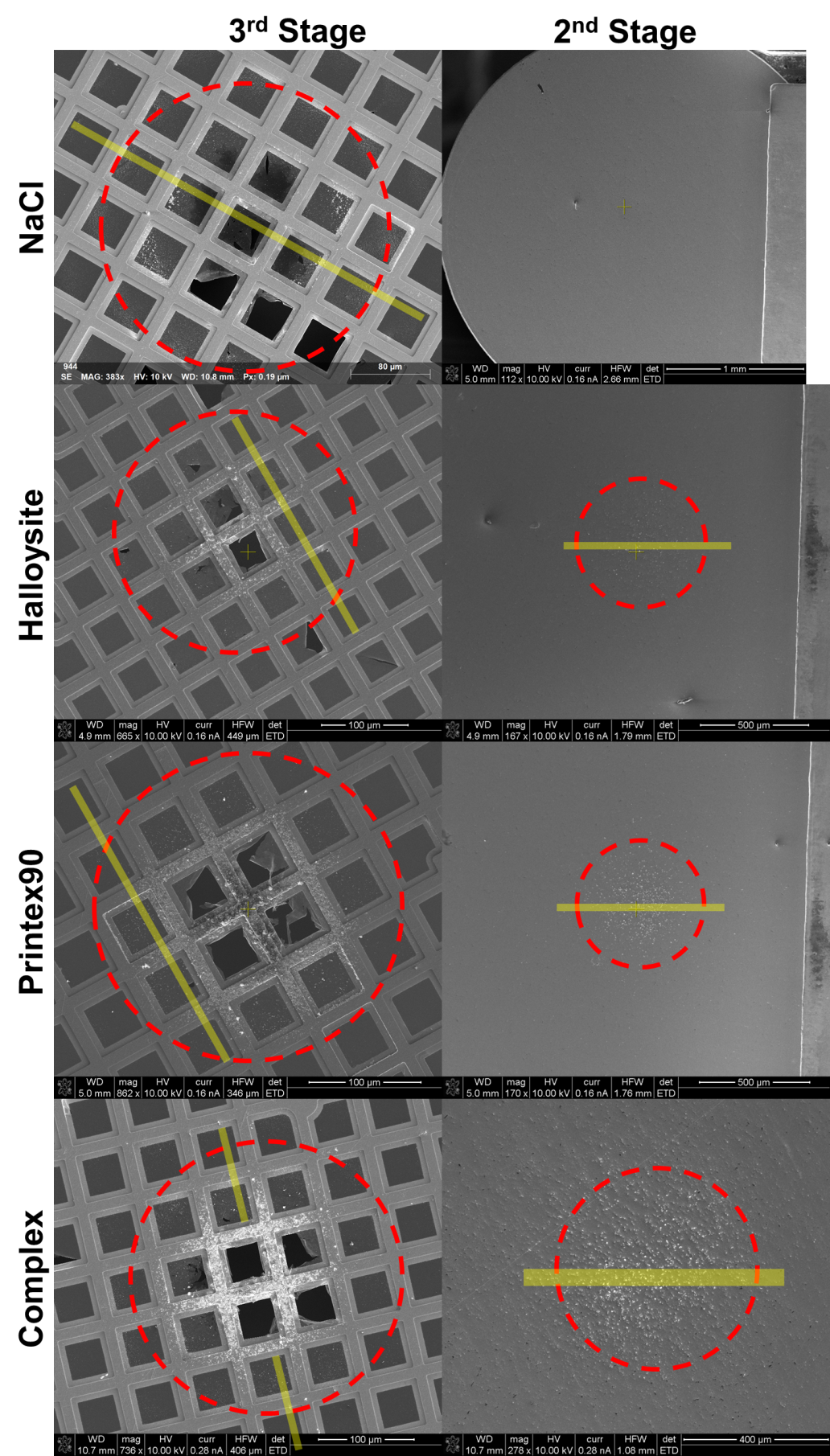


SI Figure 4. Overview images of the 2^nd^ and 3^rd^ stage impactor samples for each of the four aerosols. Red dashed circles are estimated impactor orifice positions, while the yellow lines represent the imaged areas to give a representative sample description. No impaction spot was found on the 2^nd^ stage NaCl sample so no markings were made there.

## Example images from the 2^nd^ stage of the four aerosol samples


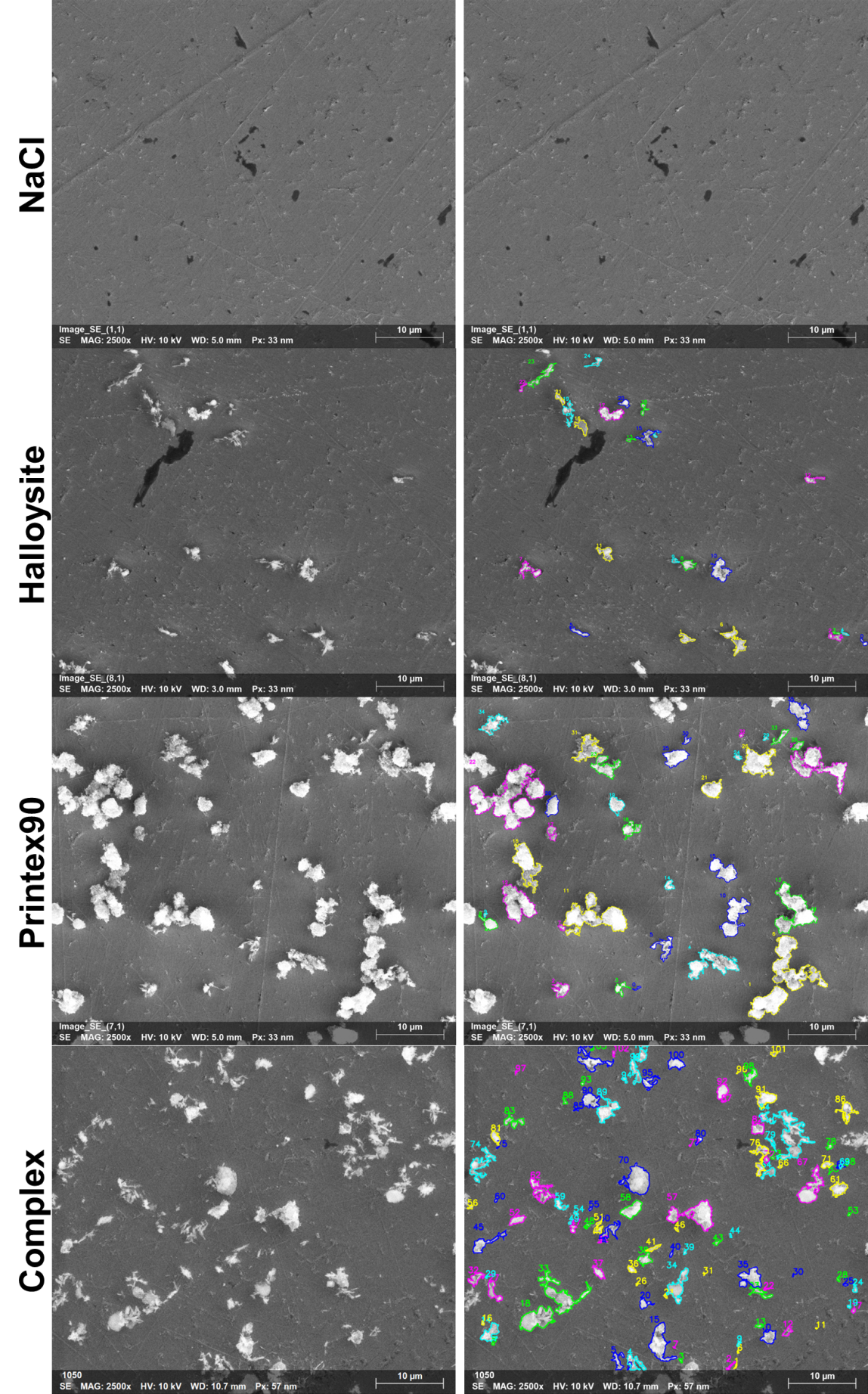


SI Figure 5. Secondary electron images from the 2^nd^ impactor stage. Original SE images (left) and segmented (right) images of the NaCl sample (top), Halloysite (second row), Printex90 (third row), and complex aerosol (bottom). Particles were collected onto TEM grids, and images were segmented with a manually set global threshold.

## Example images from the 3^rd^ stage of the four aerosol samples


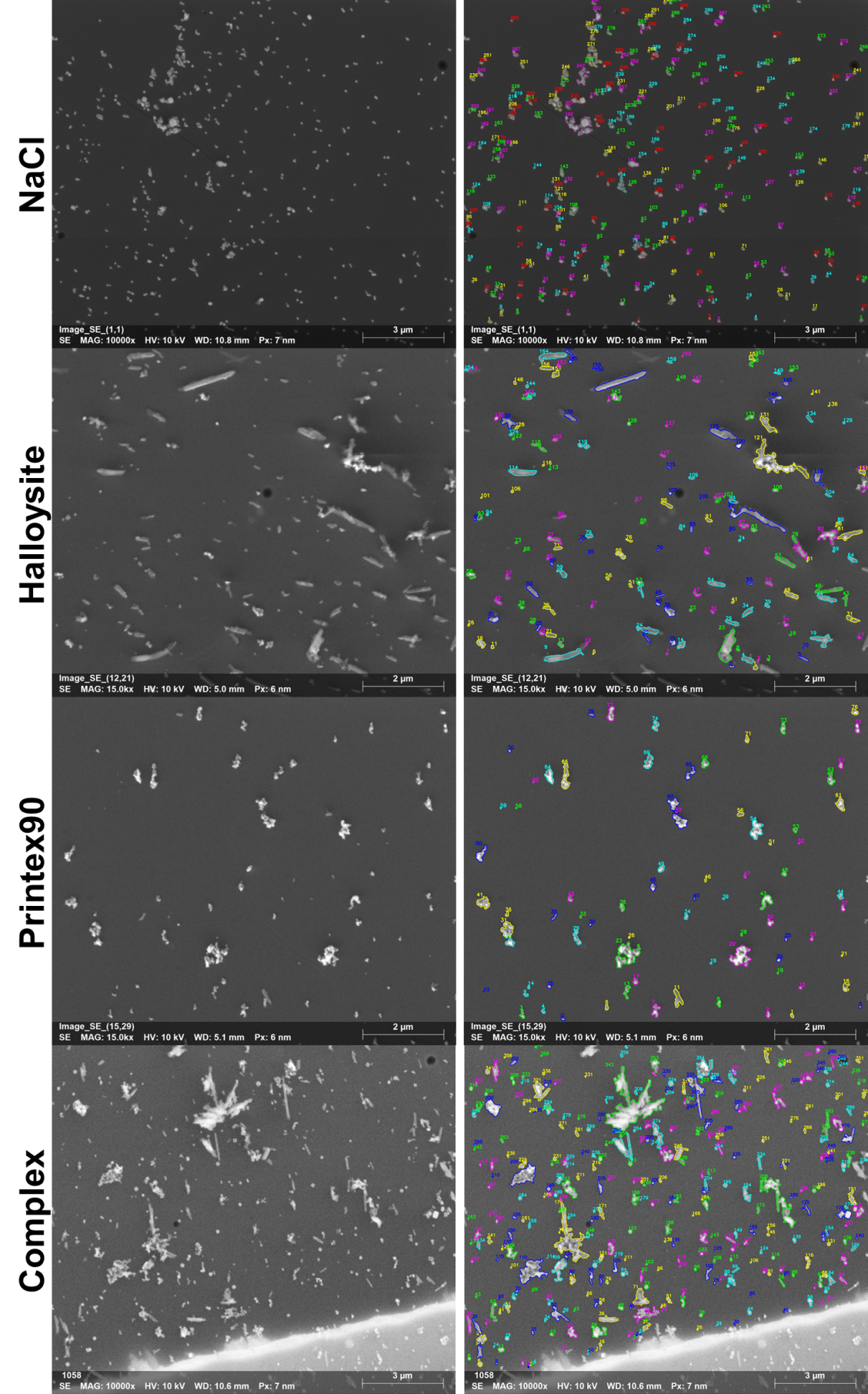


SI Figure 6. Secondary electron images from the 3^rd^ impactor stage. Original SE images (left) and segmented (right) images of the NaCl sample (top), Halloysite (second row), Printex90 (third row), and complex aerosol (bottom). Particles were collected onto Ni discs, and images were segmented with a manually set global threshold.

## Individual element maps of an area of the 2^nd^ stage complex sample


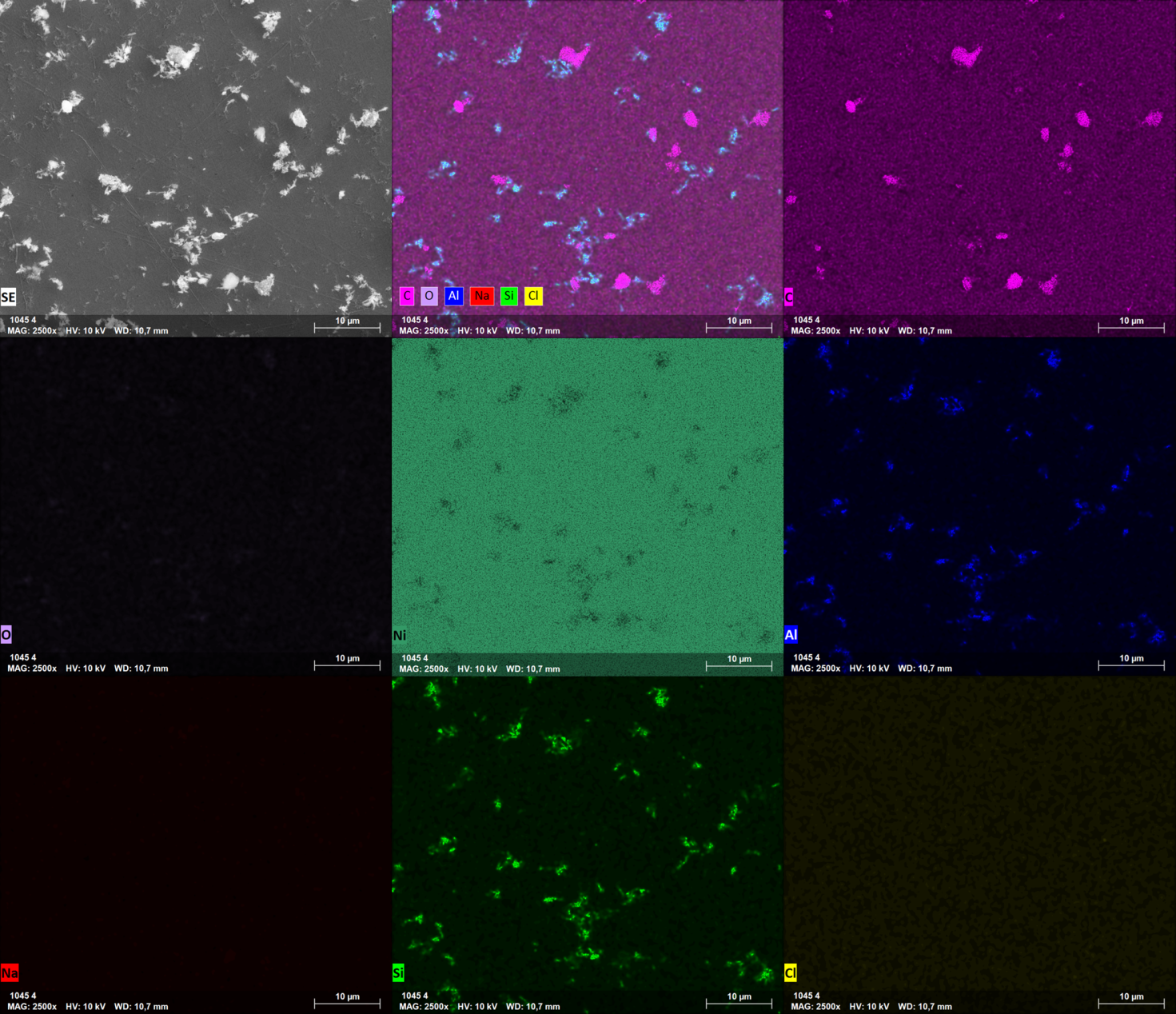


SI Figure 7. Secondary electron image (top left) of an area of the complex aerosol stage 2 sample shown along with the corresponding individual element maps of C (top right), O (mid left), Ni (mid), Al (mid right), Na (bottom left), Si (bottom mid), and Cl (bottom right), as well as an overlay of all maps (top middle). Maps were generated by summing pixel EDS spectra in 4x4 areas and converting the X-ray count in each element energy region to a normalized pixel intensity of a given color. The absolute X-ray scaling as well as the online deconvolution settings in the Esprit software were used. The Ni map is not included in the overlay of all maps, as it was found to dominate the image, making it difficult to distinguish the other elements.

## SE image of classified area


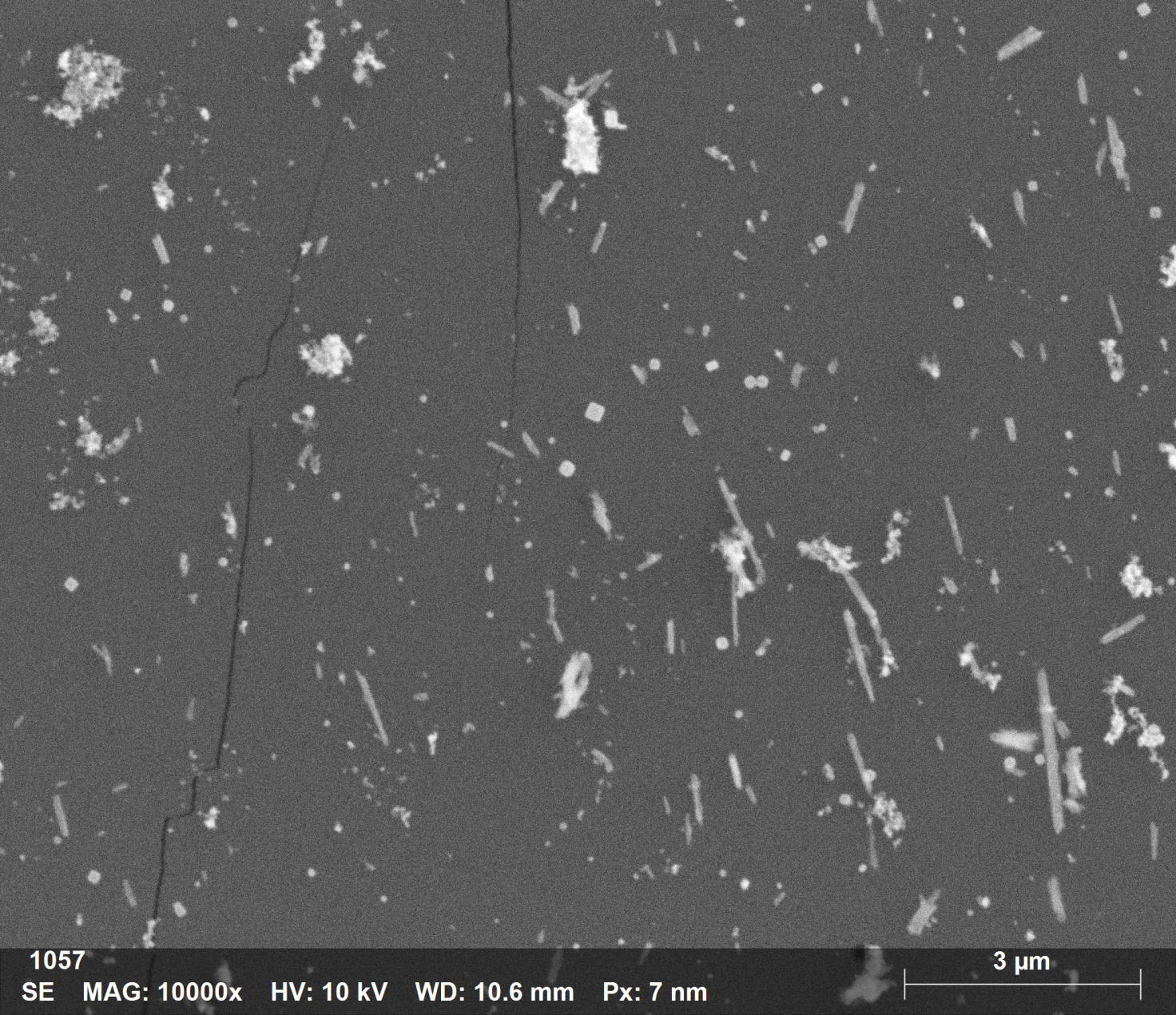


SI Figure 8. Original SE image corresponding with the maps and classified images from the 3^rd^ stage complex aerosol displayed in Figure 7.

## Class separated size distributions of the 3^rd^ stage complex aerosol


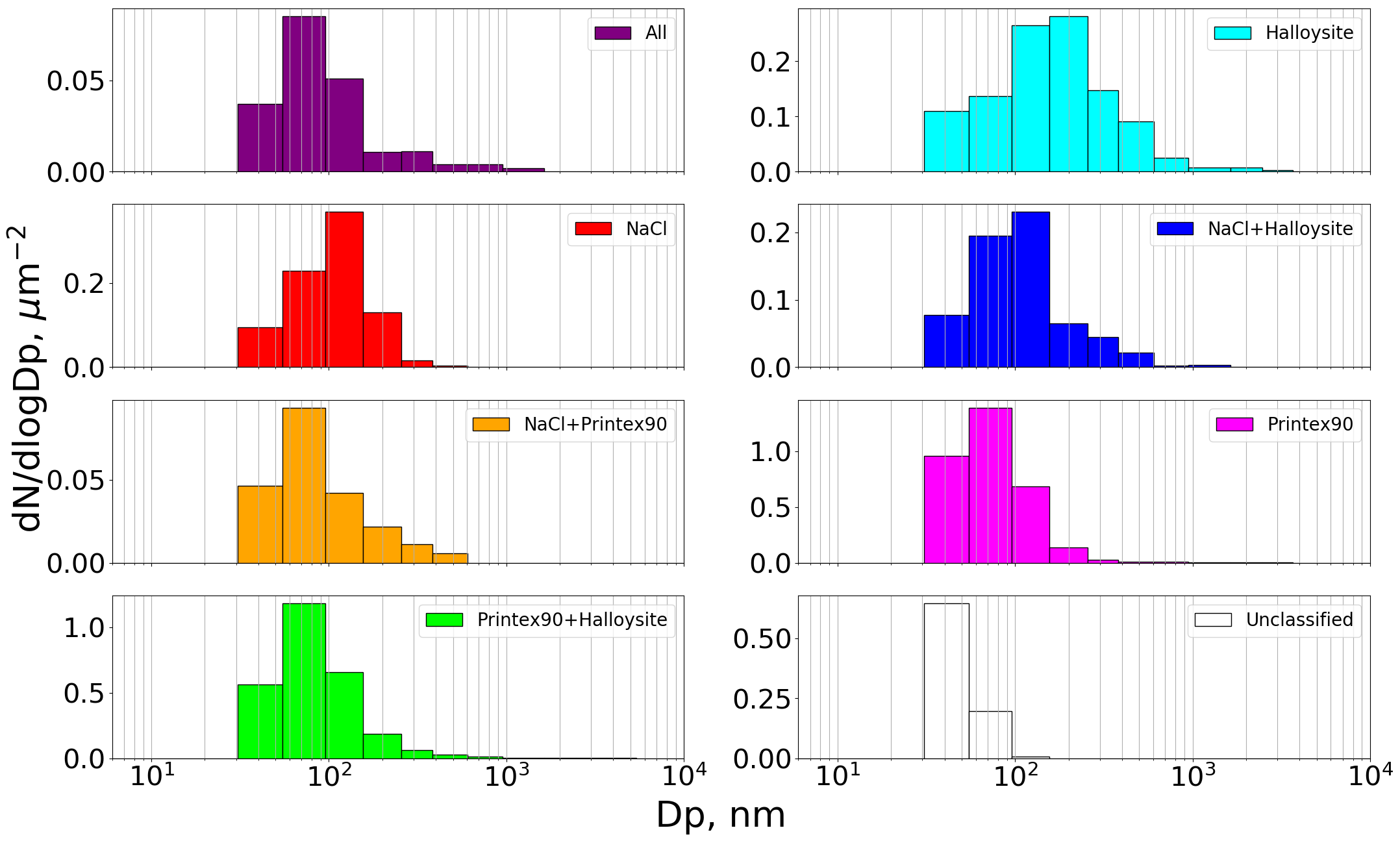


SI Figure 9. Size distributions of the individual classes identified from the 3^rd^ impactor stage of the complex aerosol sample.
